# Backbone and side-chain NMR resonance assignments of *Ciona intestinalis*$\beta \gamma$-crystallin

**DOI:** 10.1007/s12104-026-10280-7

**Published:** 2026-11-01

**Authors:** Matthew D. Jimenez, Mina Mozafari, Cat Tuong Nguyen Hoang, Miram Ali Al-Botaif, Jeongwoo Ko, Rachel W. Martin

**Affiliations:** 1 Department of Chemistry, University of California, Irvine, 1102 Natural Sciences 2, Irvine, 92697-2025 CA United States of America; 2 Department of Molecular Biology and Biochemistry, University of California, Irvine, McGaugh Hall, Irvine, 92697-3900 CA United States of America; 3 Department of Ophthalmology and Visual Sciences, Gavin Herbert Eye Institute, University of California, Irvine, CA 92617 Irvine, United States of America

**Keywords:** Crystallin, NMR, Conformational dynamics, Calcium-binding protein, Apo, Holo

## Abstract

The structural $\beta \gamma$-crystallin from the tunicate *Ciona intestinalis* serves as an evolutionary waypoint between ancestral metal cation-binding crystallins and the $\beta \gamma$ crystallins of the vertebrate eye lens. *Ci*-$\beta \gamma$ maintains the metal cation-binding sites of ancestral crystallins while also sharing common structural motifs with vertebrate lens crystallins. Due to its structure and strong interaction with Ca$^{2+}$, *Ci*-$\beta \gamma$ is more thermally and chemically stable than vertebrate lens crystallins, making it an ideal starting point for understanding $\beta \gamma$ crystallins’ evolution for molecular stability. In this study, we present the side-chain and updated backbone resonance assignments of *Ci*-$\beta \gamma$ with TALOS-N predictions on the H$_N$, H$_\alpha$, C$_\alpha$, C$_\beta$ and N chemical shifts. These assignments provide a foundation for future studies of metal binding, dynamics, and stability of proteins in the $\beta \gamma$-crystallin family.

## Biological context

The $\beta \gamma$-crystallins belong to an evolutionarily conserved protein superfamily found in organisms ranging from microbes to vertebrates. These proteins are characterized by a paired Greek key motif that assembles into a compact, stable *β*-sheet-rich fold. In vertebrates, $\beta \gamma$-crystallins function as structural proteins that maintain lens transparency and contribute to the refractive index gradient required for vision. However, homologous $\beta \gamma$-crystallins are found throughout all domains of life, where they perform a variety of biological functions (Piatigorsky and Wistow 1989; Rocha et al. 2021; Serebryany et al. 2024). Their broad phylogenetic distribution suggests that the $\beta \gamma$-crystallin fold evolved long before its specialization within the vertebrate eye lens (Slingsby et al. 2013). Many non-lenticular members of the superfamily retain metal-ion binding motifs, indicating that cation binding likely represented an ancestral function of $\beta \gamma$-crystallins prior to their recruitment as structural lens proteins.

Among extant chordates, the tunicate *Ciona intestinalis* occupies a position nearest the point in the tree of life where vertebrates diverge from the rest of the deuterostomes (Delsuc et al. 2006). As a protein from an early-diverging chordate lineage, the *Ci*-$\beta \gamma$-crystallin (*Ci*-$\beta \gamma$) preserves characteristics of both ancestral and vertebrate crystallins. *Ci*-$\beta \gamma$ contains two calcium-binding sites and retains the double Greek key motif characteristic of ancestral $\beta \gamma$-crystallin (Shimeld et al. 2005), while sharing similarities with vertebrate lens crystallins. Gene duplication of an ancestral $\beta \gamma$-crystallin gave rise to the two-domain architecture observed in modern vertebrate lens crystallins, whereas metal-binding sites were largely lost during the evolution of the lens (Ray et al. 1997; Maro et al. 2002; Suman et al. 2013). Therefore, *Ci*-$\beta \gamma$ serves as an evolutionary waypoint linking ancestral metal-binding $\beta \gamma$-crystallins with their vertebrate descendants. Previous studies showed that calcium-bound *Ci*-$\beta \gamma$ is even more thermally and chemically stable than vertebrate lens crystallins (Kozlyuk et al. 2016; Roskamp et al. 2019), which may reflect an ancestral role of the $\beta \gamma$-crystallin fold prior to its adaptation as a lens protein. In the tunicate, this protein is expressed in the sensory organ as well as the palps, suggesting dual functionality in this organism (Shimeld et al. 2005).

NMR resonance assignments are essential to characterize the biophysical basis of calcium binding, protein stability, and conformational dynamics in *Ci*-$\beta \gamma$ for both the apo and holo forms. Furthermore, backbone chemical shift assignments provide information regarding local secondary structure propensities and establish a framework for future investigations of metal-dependent structural changes, backbone dynamics, and intermolecular interactions. Here, we report the updated backbone and side-chain resonance assignments of *Ci*-$\beta \gamma$. HNCO, HN(CO)CA, HNCA, HN(CA)CO, CBCA(CO)NH, and HNCACB experiments were performed to assign the backbone. HN, N, C*α*, and C*β* chemical shifts were obtained for all non-proline residues for both the apo and holo forms. Unambiguous H$_\alpha$ and H$_\beta$ assignments were obtained for 78/84 residues by performing a $^{1}$H-$^{15}$N HSQC and a 3D HBHACONH experiment. Extensive side-chain assignments, including C$_\gamma$, C$_\delta$, and C$_\epsilon$, were also measured using a 3D CCCONH experiment. Backbone assignments were used to predict secondary structure (SS) and calculate random coil index-predicted S$^{2}$ order parameters (RCI-S$^{2}$) with TALOS-N (Shen and Bax 2013). These assignments establish a foundation for future investigations of calcium-dependent structural stability, conformational, dynamics, and molecular evolution within the $\beta \gamma$-crystallin lineage.

## Methods and experiments

### Expression and purification of *Ci*-$\beta \gamma$-crystallin

An *Escherichia coli* codon-optimized plasmid containing the *Ci*-$\beta \gamma$-crystallin gene was purchased from Blue Heron Biotechnology (Bothell, WA). The protein sequence is

MGKIILFEDV EFGGKKLELE TSVSDLNVHG FNDIVSSIIV ESGTWFVFDD EGFSGPSYKL TPGKYPNPGS WGGNDDELSS VKQQ. Expression of *Ci*-$\beta \gamma$ for the side-chain NMR experiments was performed as follows: bacteria containing a pET-28 plasmid with the *Ci*-$\beta \gamma$ sequence and a kanamycin resistance gene were plated on a kanamycin (IBI scientific, Dubuque IA USA) Luria-Bertani (LB) agar plate at 37$^{\circ }$C for 16 h. An overnight culture was inoculated with a colony from the *Ci*-$\beta \gamma$ plate and incubated at 37$^{\circ }$C for 12–16 h. 500 mL of kanamycin LB media was inoculated with 5 mL of overnight culture and incubated at 37$^{\circ }$C until OD 1.1 was reached. The media was then spun down at 4,000 RPM for 30 minutes at 4$^{\circ }$C. The supernatant was discarded, and the pellet was resuspended in $^{13}$C/$^{15}$N labeled media and incubated at 37$^{\circ }$C for one hour. It was then induced with 500 *μ*L of 1 mM IPTG (Goldbio, St. Louis, MO USA) and incubated at 16$^{\circ }$C for 48 hours. The media was then spun down and flash frozen for storage. To purify *Ci*-$\beta \gamma$, cell pellets were resuspended in 10 mM BisTris (Promega, Madison WI USA), 0.05% NaN$_{3}$ (Fisher Bioreagents, India), 10 mM CaCl$_{2}$ (Fisher chemical, Germany), pH 7 and lysed with sonication. The lysate was then purified via one anion exchange and one run of size exclusion chromatography (SEC). The low salt buffer utilized was 10 mM BisTris, 0.05% NaN$_{3}$ at pH 7 and the high salt buffer was 10 mM BisTris, 1 M NaCl, 0.05% NaN$_{3}$ at pH 7 for anion exchange. The buffer utilized for SEC was 10 mM HEPES (Fisher Bioreagents, Taiwan), 150 mM NaCl (Spectrum Chemical MFG Corp, New Brunswick NJ), and 0.05% NaN$_{3}$ at pH 7. Purity was checked through mass spectrometry and gel electrophoresis. Sample preparation for backbone chemical shift assignments was performed using a similar procedure as previously described (Kozlyuk et al. 2016).

### NMR measurements and data analysis

Experiments for the backbone assignments were performed at 25$^{\circ }$C on a Varian $^{Unity}$INOVA system operating at 800 MHz equipped with a $^{1}$H/$^{13}$C/$^{15}$N 5 mm tri-axis PFG triple-resonance probe. The protein concentrations were 1.7 mM in the presence of 10 mM Tris (pH 7.1), 0.05% NaN$_{3}$ and 10 mM EDTA or 10 mM CaCl$_{2}$. HNCO, HN(CO)CA, HNCA, HN(CA)CO, CBCA(CO)NH, and HNCACB experiments were acquired to assign the protein backbone. All the data for the backbone assignments were processed in NMRPipe and sparky. The assignments were performed in CcpNMR. Experiments for the side-chain assignments were performed at 25$^{\circ }$C on a Bruker AVANCE NEO 800MHz spectrometer equipped with a BCU II chiller and a 5 mm $^{1}$H/$^{13}$C/$^{15}$N inverse triple resonance probe. The protein concentration for the side chain experiments was 0.5mM in 10mM HEPES (pH 7.1) 50mM NaCl, 0.05% NaN$_{3}$ with 10mM EDTA or 10mM CaCl$_{2}$. HBHA(CO)NH, $^{1}$H-$^{15}$N HSQC, $^{1}$H-$^{13}$C HSQC, CC(CO)NH, and a hCCH-TOCSY were acquired to assign the protein side-chains. All the data for the side-chain assignments were processed in TopSpin v 4.3 and the assignments were assigned on CcpNMR. The parameters for all NMR experiments can be found in the supplementary information page.

### Secondary structure calculation

**Fig. 1:**
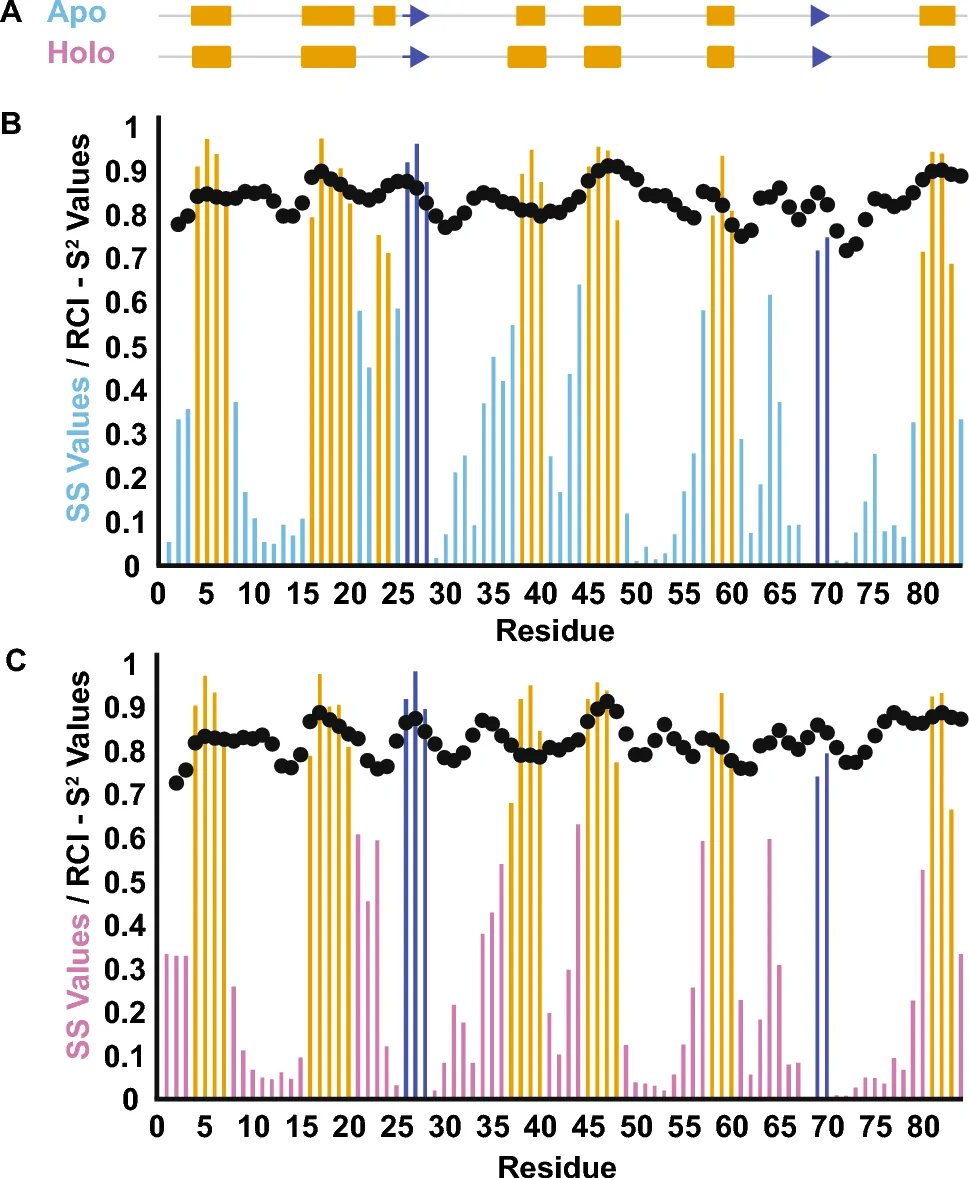
The *Ci*-$\beta \gamma$ secondary structure (SS) and random coil index-predicted S$^{2}$ order parameter (RCI-S$^{2}$) TALOS-N predictions for both apo (treated with 10 mM EDTA) and holo (treated with 10 mM CaCl$_{2}$) forms. **A** Predicted *β*-sheet secondary structure (orange) and *α*-helix secondary structure (blue) from the TALOS-N prediction. **B** RCI-S$^{2}$ and secondary structure prediction of the apo form of *Ci*-$\beta \gamma$. **C** RCI-S$^{2}$ and secondary structure prediction of the holo form. Both measurements show a difference in *β* sheet-secondary structure when Ca$^{2+}$ is bound

TALOS-N calculations were performed for both the apo and holo forms of *Ci*-$\beta \gamma$ using these backbone assignments. In the apo but not the holo form, chemical shift analysis indicates that Val 23, Ser 24, and Ser 80 are involved in *β*-sheet secondary structure. In contrast, Ser 37 is in a *β*-sheet secondary structure in holo form but not the apo form (Fig. 1A). These results indicate that there are localized differences in secondary structure when calcium is bound. Residue Val 23 and Ser 24 exhibit reduced *β*-strand character and lower predicted RCI-S$^{2}$ values, suggesting an increase in local flexibility. Ser 37 exhibits *β*-strand character when calcium is bound with minimal differences in S$^{2}$, indicating that there is subtle rearrangement in the backbone upon calcium binding. Ser 80 shows minor differences in both *β*-strand character and S$^{2}$, demonstrating that calcium binding has little effect on its local rigidity (Fig. 1B and C). Overall, calcium binding does not appear to induce major refolding of *Ci*-$\beta \gamma$, but instead redistributes local conformational populations and alters secondary structure in specific regions. The loss of *β*-strand character at Val 23, Ser 24, and Ser 80, with the increased *β*-strand character at Ser 37 likely reflects a subtle calcium-induced change in the backbone conformation and hydrogen-bonding interactions.

**Fig. 2:**
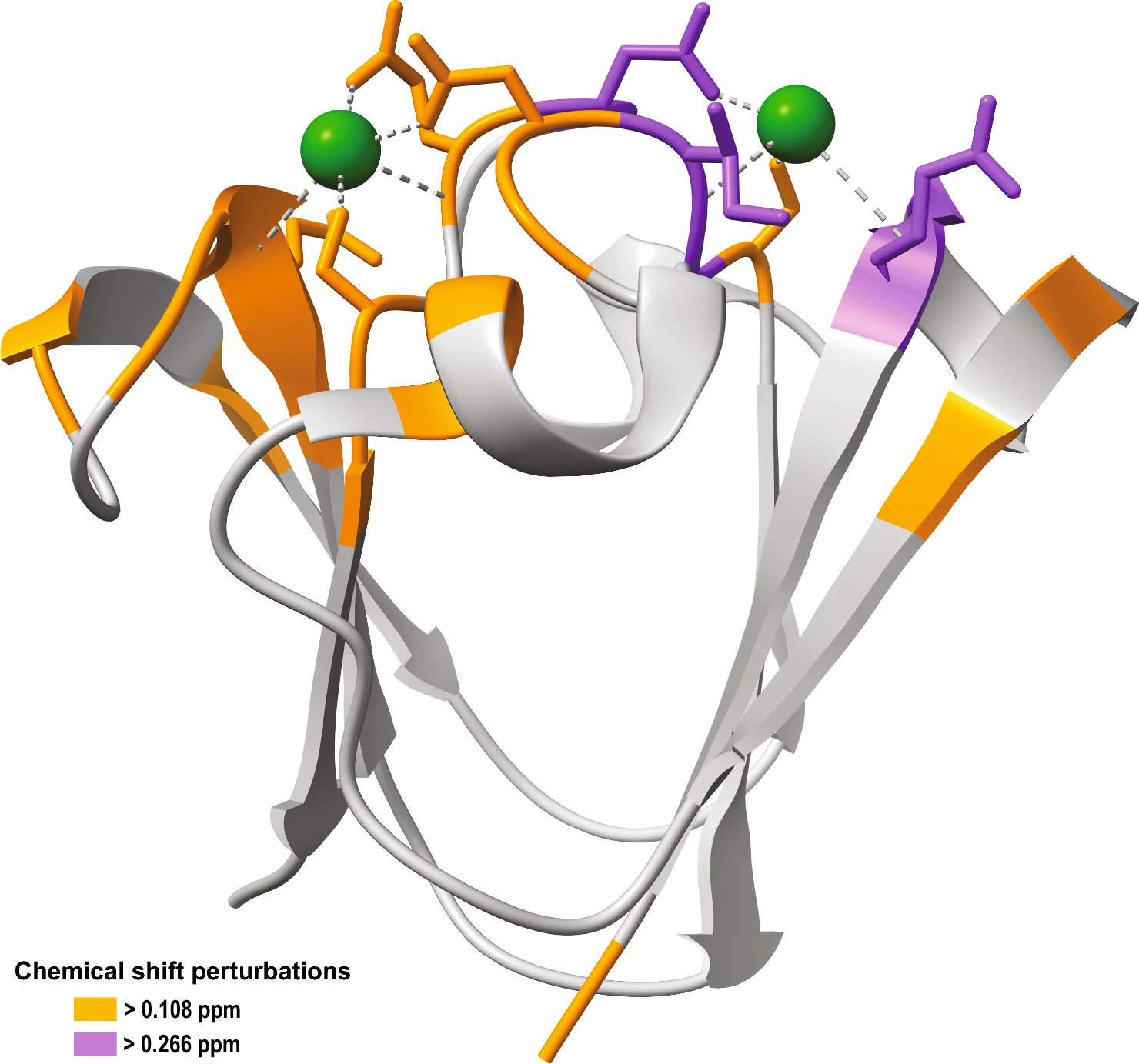
Mapping of calcium ion-dependent chemical shift perturbations onto the *Ci*-$\beta \gamma$ structure. Chemical shift perturbations (CSPs) calculated from apo-to-holo amide $^{1}$H-$^{15}$N chemical shift differences were mapped onto the structure of *Ci*-$\beta \gamma$-crystallin (PDBID: 2BV2) (Shimeld et al. 2005). Residues with CSPs greater than the mean (0.108 ppm) are shown in orange, while residues with CSPs greater than the mean plus one standard deviation (0.266 ppm) are shown in purple. The largest perturbations are concentrated within one calcium-binding site, with perturbations observed at nearby non-coordinating residues

**Fig. 3:**
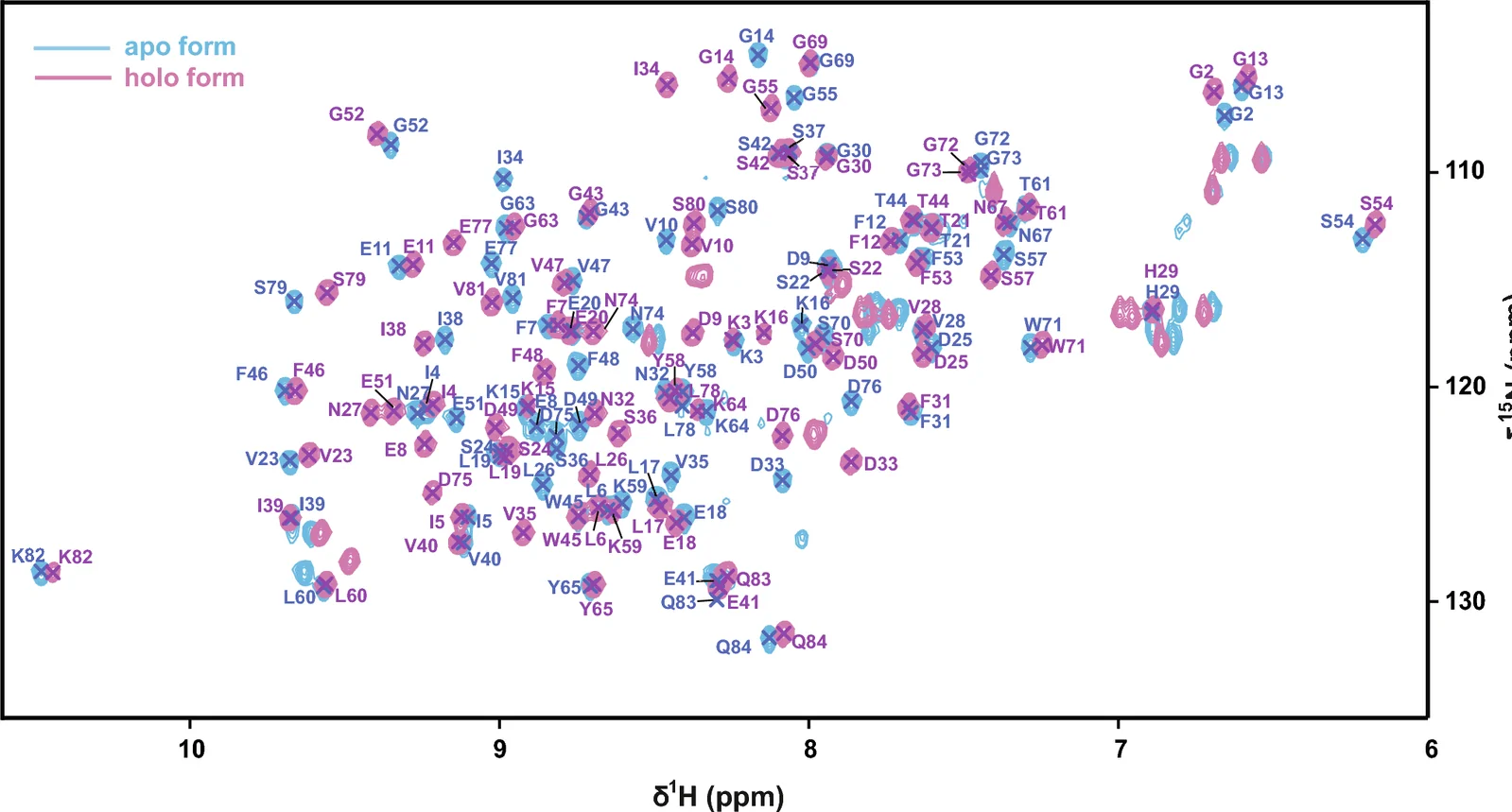
$^{1}$H-$^{15}$N HSQC of the apo (treated with 10 mM EDTA) and holo (treated with 10 mM CaCl$_{2}$) forms of *Ci*-$\beta \gamma$ at 298 K. The cross peaks assignments are labeled with their respective one letter residue codes and sequence numbers

Comparison of TALOS-N predictions with the crystal structure revealed similar secondary structure, although several residues near secondary structure boundaries showed different assignments, potentially reflecting differences in conformation and dynamics between a crystal structure and a molecule in solution. To further assess the regions most affected by calcium binding, apo-to-holo $^{1}$H-$^{15}$N chemical shift differences were mapped onto the *Ci*-$\beta \gamma$ structure. The largest chemical shift perturbations (CSP) near the calcium-binding sites, with particularly pronounced changes observed for residues Asp 9, Ile 34, Val 35, Ser 36, Asp 75, and Asp 76. The residues with the largest CSPs are found in one binding site suggesting that the two sites are experiencing different local structural or chemical environments upon calcium binding. Perturbations were also observed for nearby non-coordinating residues, which suggests that calcium binding affects the local structural environment surrounding the calcium-binding sites as well (Fig. 2). Together, these chemical shift changes indicate that calcium binding produces localized structural and environmental changes that extend beyond the directly coordinating residues (Fig. 3).

## Extent of assignment and data deposition

Excluding the four proline residues, the overall backbone assignment completeness was 100% for both apo and holo form of *Ci*-$\beta \gamma$. Backbone chemical shift assignments were deposited in the BMRB under accession numbers 26860 (apo) and 26861 (holo). The backbone assignments for both the apo and holo forms are labeled on overlaid $^{1}$H-$^{15}$N HSQC spectra (Fig. 1). Side-chain assignments were obtained for 96.3% of both apo and holo forms. The chemical shifts for C$_\gamma$, C$_\delta$, and C$_\epsilon$ resonances were assigned for 79 out of 82 possible side chain carbons. In the apo form, the unassigned nuclei were Ile 39 C$_\gamma 2$, Thr 61 C$_\gamma 2$, and Gln 84 C$_\gamma$. In the holo form, the unassigned nuclei were Ile 34 C$_\delta 1$, Thr 61 C$_\gamma 2$, and Gln 84 C$_\gamma$. The missing Ile 34 C$_\delta 1$, Ile 39 C$_\gamma 2$, and Thr 61 C$_\gamma 2$ resonances can be attributed to spectral overlap or line broadening potentially arising from local dynamics. The Gln 84 C$_\gamma$ resonance could not be assigned because it is the C-terminal residue and lacks the sequential connectivity required by the side-chain assignment experiments utilized during this study. Side-chain chemical shift assignments were deposited in the BMRB under accession numbers 53880 (apo) and 53882 (holo).

Overall, we report near complete backbone and side-chain resonance assignments for both the apo and holo form of *Ci*-$\beta \gamma$. The comparison of the apo and holo chemical shifts demonstrates that calcium binding does not produce large scale structural rearrangements, but instead results in localized changes in *β*-strand propensity and backbone rigidity. TALOS-N analysis indicates differences in secondary structure surrounding residues involved in calcium binding which supports a model in which calcium stabilizes specific regions of the protein while preserving the $\beta \gamma$-crystallin fold. These assignments provide a comprehensive NMR framework for investigating the molecular basis of calcium-dependent stability, conformational dynamics, and metal binding in *Ci*-$\beta \gamma$. Due to *Ci*-$\beta \gamma$ evolutionary significance of being a evolutionary intermediate between ancestral $\beta \gamma$-crystallin and vertebrate lens crystallins, this data has established an important foundation for future structural, biophysical, and evolutionary studies aimed at understanding how the $\beta \gamma$-crystallin fold adapted from a calcium ion-binding protein to a specialized structural protein in the vertebrate lens.

## Supplementary information

Raw NMR files with parameters can be found in BMRB 53880 (apo) and 53882 (holo).

## Data Availability

Backbone chemical shift assignments were deposited in the BMRB under accession numbers 26860 (apo) and 26861 (holo). The side-chain chemical shift assignments were deposited in the BMRB under accession numbers 53880 (apo) and 53882 (holo).
